# Taking alcohol by deception: an analysis of ethanol concentration of "paraga" an alcoholic herbal mixture in Nigeria

**DOI:** 10.1186/1756-0500-5-127

**Published:** 2012-03-06

**Authors:** Oluwadiya S Kehinde, Akinola E Adegoke

**Affiliations:** 1 Department of Surgery, College of Medicine, Ekiti State University, Ado-Ekiti, Ekiti, Nigeria; 2 Central Science Laboratory, Obafemi Awolowo University, Ile-Ife, Osun, Nigeria

## Abstract

**Background:**

Alcohol related road traffic injuries are on the rise in Nigeria. A sizable proportion of the alcohol intake is disguised as herbal medicines which are commonly available at motor parks in most urban centres. This study aims to determine the ethanol concentration of the herbal preparations and the vendors' knowledge about their preparation and use. Twenty-eight samples of the paraga mixtures were obtained for analysis from 22 paraga vendors. The vendors were interviewed in the motor parks using a semi-structured questionnaire.

**Results:**

All the paraga outlets were located in or near motor parks. Commercial motor drivers and motorcyclists accounted for most customers. There were no formal recipes, production involved no calibrations or weighing and thus the components and concentration of different batches varied. The alcohol by volume (ABV) of the samples ranged between 1.20% and 20.84%. Nine samples were weaker than beers (Alcohol By Volume (ABV) of 1-3.1%). Ten were equivalent to beer (ABV:3-8%) and the rest were equivalent to wine (ABV:8-12%) or stronger (ABV: 18-20%).

**Conclusions:**

Paraga should be classified as alcoholic beverages, and its sale restricted as such. The production should come under scrutiny, because the haphazard ways they are prepared may pose other health risks apart from those due to their alcoholic contents.

## Background

Globally, alcohol is responsible for 1.8 million (3.2%) deaths annually or 4.0% of the global disease burden. About 50% of these deaths are due to injuries [1,2]. While alcohol related fatalities are decreasing in high-income countries (HIC) [3], the increase in alcohol related problems in Low-middle income countries (LMIC) is alarming [1,4]. Alcohol has been implicated in a large proportion of road crashes, and what makes it more poignant is that many other people apart from the drunk driver are injured or maimed [5-9]. This secondary effect can only be magnified in LMIC countries where buses, mini-buses and motorcycle taxis are the predominant means of transportation [10-12].

While alcohol remains the dominant drug causing impairment of driving performance, other drugs, especially in combination with alcohol, increase collision risk [13]. The effects of these drugs are not as well understood as the effect of alcohol [14]. Studies in Britain revealed a 6% incidence of medicinal drug use in fatal road crashes [15]. The incidence also seemed to be increasing in Denmark and other HIC [16]. To our knowledge, there are no formal studies in Nigeria showing effects of medicinal drug usage on driving, but various studies have alluded to the use of alcohol in making herbal medicine in Nigeria [17,18].

In Nigeria, one of the most easily accessible forms of medicinal herbs is paraga with the following synonyms: opa-ehin, foganna and fidigbogi. Paraga is an herbal mixture with assorted ingredients and indeterminate alcohol content. It is popular in many neighbourhoods in Nigeria. It is popularly used as a stimulant and is believed to have curative effects on an extraordinary range of ailments which may be the reason why it is so popular among the populace [19]. It is commonly sold by vendors in motor parks where commercial drivers and motorcyclists have easy access to them [19].

This is the first of a three-part study of paraga and its role in road safety in Osogbo in southwest Nigeria. This paper reports the paraga vendors' knowledge of paraga composition and production as well as determines the alcoholic content of samples of paraga obtained from vendors of the drink located within and around motor parks in Osogbo, Osun State Nigeria. The findings from this study can serve as an objective and logical basis for promulgating policies on paraga consumption especially among drivers and motorcyclists.

## Methods

The survey was conducted in Osogbo, the capital of Osun State in southwest Nigeria. It is a nodal town with road connections to surrounding towns and other cities in the country. Osogbo has seven motor parks for intercity drivers and they were the locations of the survey. Each motor park consists of a large open space surrounded by kiosks and shops where food stuff, drinks including herbal mixtures, and other items were sold.

The target population for this study was the paraga vendors in the motor parks. The vendors were interviewed in the motor parks using a semi-structured questionnaire, which contained questions about the composition of the herbal mixtures, how the mixtures were made, who made them and the vendors' usual hours of operation. Written informed consent was obtained from all respondents. Samples of the mixtures were also taken for analysis of ethanol content. Analysis was by the second author at the Central Science Laboratory of the Obafemi Awolowo University Ile-Ife using colorimetric method [20]. We used SPSS version 15 for the frequency analysis of the data.

Ethical permission for the study was obtained from the Ethical Committee of the Ladoke Akintola University of Technology (LAUTECH) Teaching Hospital Osogbo, Nigeria.

## Results

Of the twenty nine vendors that were identified selling paraga at or near motor parks, twenty-two (75.9%) agreed to be interviewed. Table 1 shows some sociodemographic characteristics of the hawkers. Twelve of them (54.5%) were employed in some other occupations such as trading (seven (31.8%) and barbing; in fact, one of them was also moonlighting as a driver. Twelve (54.5%) start selling paraga by 6 am, and by 8 am; all of them have begun selling. Nineteen (86.4%) had their briskest sales in the morning, one (4.5%) in the evenings and two (9.1%) during the night. Hardly any sales were made in the afternoons, and so most vendors--especially those with other jobs--do not sell paraga in the afternoons. Raining season was cited by 18 (81.8%) as the season when they usually made the best sales, followed by Harmattan and the dry season, each cited by two vendors.

**Table 1:** Some characteristics of paraga vendors

Attribute	Number	Percentage
Sex		
Female	17	77.3
Male	5	22.7
Religion		
Islam	16	72.7
Christian	6	27.3
Education		
None	1	4.5
Primary	7	31.8
Secondary	13	59.1
Other occupations		
Has other jobs	12	54.5
Has no other jobs	10	45.5

### Paraga vendors' experience and knowledge about the making of paraga

The vendors have been selling paraga for between 1 and 20 years (Median: 3 years). Twenty (90.4%) sold the preparation from fixed locations such as stands (15 (68.2%), Kiosks (three (13.6%) or shops (two (9.1%); while the remaining two were itinerant. Fourteen (63.6%) usually made the herbal preparation themselves, 6 (27.3%) purchased their wares from other producers while the remaining 2 (9.1%) usually procure their stock from herbal wholesalers. While there was a great assortment of paraga on display by some of the vendors, the majority of them had just one to four varieties to display (Table 2). When asked the question, "To your knowledge, approximately, how many of these brands contain alcohol?" the responses showed that most of the brands on sale contained alcohol. Thirteen (59%) vendors admitted to having only alcohol-containing brands of paraga for sale.

**Table 2:** Varieties or brands of paraga on sale by the vendors

Variety (brands)	Number of vendors	Percentage
1-4	11	50.0
5-6	2	9.1
7-8	4	18.2
9-10	1	4.5
11-12	1	4.5
13-14	2	9.1
> 15	1	4.5
Total	22	100.0

The most common ingredients used in making paraga were herbs, followed by alcohol in 77% of the vendors (Table 3). Lime and pineapple were also commonly used. When the investigators enquired about how the samples purchased for laboratory analysis could be stored, the vendors specifically instructed them not to store some of the samples which contained lime and pineapple, in the refrigerator because "cold will spoil their potency".

**Table 3:** Ingredients used in making paraga

Ingredient	Number of vendors using ingredient
Herbs	22 (100)
Alcohol	17 (77.3)
Water	14 (63.6)
Lime	14 (63.3)
Minerals (eru (Sylopia acthipea), aayu (garlic (Alium satirum), iyere (African black pepper)	8 (36.4)
Pineapple	6 (27.3)
Others (ginger (Singuber officinale), onion leaves (Allium ascabricum), and animal horn/hoof, orogbo (bitter kola)	5 (22.7)

Most vendors were unwilling to describe the process of manufacturing their own brand of paraga, and only four vendors volunteered to give a detailed description of the process involved:

"I mix herbs e.g. kannafuru (cloves), kaun (trona); eeru (Sylopia acthipea), kafura (camphor); alubosa elewe (onion, Allium cepa and Allium ascabricum) together, arrange them in bottles and soak them in any kind of alcohol..."

"Some kind of herbs including ginger (Singuber officinale), kannafuru, and kooko oba (African lemon grass), will be cut into square pieces and arrange in a clean bottle. Then I pour any type of alcohol into the bottle."

"I mix and arrange eru (Sylopia acthipea), aayu (garlic (Alium sa tirum), iyere (African black pepper), kaun (trona), kafura (camphor), and other ingredients in a bottle and then pour either water or any of alcohol brand into it."

"Combine and arrange (sic) of the above mentioned ingredients in a bottle soaked with any alcohol of your choice."

Thus, there were no specific recipes, the processes involved no calibrations or weighing of ingredients; therefore, the components and concentrations of different batches varied.

### Paraga vendors' knowledge of the reasons for their customers' patronage

Twenty (90.9%) believed the main reason why their customers patronize them was medicinal and only two (9.1%) believed that it was for refreshments. The vendors who believed that paraga was medicinal were asked to list the three most common medical reasons for which their wares were patronized and the results are shown in Table 4. The number of patrons catered to per day by the vendors were < 25 (five vendors), 56-50 (eight vendors), 51-75 (six vendors) and > 100 (three vendors). When asked to suggest the three most common group of people who patronized them, drivers were cited 20 (39.2%) times, bricklayers (self-employed building construction workers), 13 (25.5%) times, commercial motorcyclists, 10 (20%) times, mechanics 7 (13.7%) times and "educated people" once.

**Table 4:** Medicinal purposes of paraga as suggested by the vendors

Medicinal purpose	Number of times (%)
Back pain	17 (37.0)
Fever	12 (26.1)
Dysentery	8 (17.4)
Pile	5 (10.9)
Weak erection	3 (6.5)
Gonorrhea	1 (2.2)

Eighteen (81.8%) vendors had taken the products that they were selling while four claimed they had not, for reasons like: "I am spiritual", "I just didn't like it" and "known only to me". Fourteen (63.6%) believed that paraga could make a person drunk, 7 (31.8%) believed it could not while one (4.5%) respondent did not know. Fourteen (63.6%) had seen people get drunk after drinking paraga while eight (36.4%) had not.

### Analysis of alcohol contents of paraga

The alcohol content of drinks is most commonly expressed as Alcohol by Volume (ABV), and is a standard measure of how much ethanol is present in an alcoholic beverage. It is expressed as a percentage of total volume. Twenty eight samples of paraga were taken. Their alcoholic content ranged between 1.20% to 20.84% alcohols. Nine (32.1%) had ABV of 1-3%, ten (35.7%) had ABV of 3-8%, six (21.4%) had ABV of 8-12% while the last three (10.7%) had ABV of 12-20%

## Discussion

Since time immemorial, it has been widely believed in most part of Sub-Saharan Africa that alcohol has medicinal properties [21,22]. These curative properties were believed to be accentuated by combining medicinal herbs and alcohol. This mixture, called *agbo *in Yorubaland is traditionally dispensed by native doctors as prescriptions for diseases and ailments. However, over the past 15 years, paraga or opa-ehin, a more popular, easily accessible and more potent form of native concoction has become popular especially in the southern part of Nigeria.

Our study showed that all the paraga samples obtained for analysis were alcoholic. Globally, there are three main kinds of alcoholic beverages: beers which normally contain from 3 to 8 percent alcohol; wines contain from 8 to 12% alcohol and distilled spirits such as whiskey, gin, or vodka, contain about 40 to 50% alcohol. More than two-thirds of the samples had ethanol strength that was either equal to or stronger than beers. Since our samples were freshly brewed and were kept in the refrigerator until analyzed, the values we obtained might be the lowest obtainable values, for as Partanen observed, "traditional beverages are consumed" live," i.e., in a state of continuing fermentation" [9]. This view is buttressed by the advice given by the vendours that we should not refrigerate some of the samples so that their potency would not be lost. Alcohol has an important effect on driver behaviour and performance. These effects start at the lowest measurable level and increases as the Breath Alcohol Concentration (BAC) level increases [13]. There is no evidence for a particular threshold value above which there is a transition from unimpaired to impaired [13]. Impairment is not the same as drunkenness. Impairment of cognitive and sensory function, which is necessary for skilful driving, starts much earlier than before intoxication becomes evident. Excessive alcohol intake can also affect other parts of the body. There is a strong correlation between excessive alcohol use and an increased risk of developing alcoholic liver disease, cardiovascular disease, malabsorption, chronic pancreatitis, and some form of cancer. According to the extant laws in Nigeria, the ethanol content of paraga classifies it as an alcoholic beverage [18]. There is a law in place for both an off and on-license regulation for alcohol in Nigeria, which also restricts the hours of sales of alcohol. The law extends to all classes of alcoholic drinks including spirits and wines. But this law is poorly enforced for wine and beers and disregarded for medicinal preparations [23]. It is therefore important that paraga should be classified as alcoholic beverages, and its sales restricted as such.

It is alarming that majority of the vendors were located in or close to motor parks because studies have shown that making alcohol available in certain outlets is associated with increased rates of alcohol-related problems such as drunk driving [23]. Currently the alcohol control policy in Nigeria does not ban the consumption of alcohol in public places such as motor-parks and in public transports [23].

The popularity of traditional herbal remedies has generated concern about their safety among health authorities [24,25]. The finding from this study that most vendors have no formal recipe for making paraga showed that control measures for ensuring quality as well as consistency in formulation is poor. Phrases like "some kinds of herbs" and "soaked in any kind of alcohol" showed that paraga makers had no strict rule guiding what ingredients are used, and the quantity used to manufacture the beverage. An even more alarming issue is the potential toxicity of the constituents to the consumers. Producers of alcoholic beverages have been known to deliberately include poisons in their wares in other to make them more potent or reduce production costs [23]. This has been reported in India where drinkers of illicit alcoholic beverages have been poisoned by methanol and other contaminants [23]. There are numerous newspaper articles and anecdotal reports of paraga containing cannabis being sold in Lagos, South-Western Nigeria [26]. The potential long term health effect of paraga on health is unknown, but other medicinal herbs in Nigeria have been shown to contain elevated level of toxic substances: Obi et. al. had in a previous study of 25 brands of herbs obtained from all over the country showed all samples had elevated levels of heavy metals [25,27]. There have also been newspaper reports of people dying from paraga poisoning [27].

## Conclusions

Our study provides incontrovertible evidence that paraga is an alcoholic beverage. We have also shown that paraga is haphazardly concocted; the production processes were not structured, thus there are potentials for contamination by both chemical and biological agents. Finally, paraga vendors are mostly found at or near motor packs and more than 50% of their customers were commercial drivers and motorcycle riders.

Considering the prevalence of alcohol related road traffic injuries in Nigeria, a policy to restrict access to all alcoholic beverage (including paraga) in motor and motorcycle parks across the country should be put in place and strictly enforced. A sustainable health education campaign should be established in motor and motorcycle parks to educate drivers and motorcycle riders on the harmful effects of paraga. The health education should emphasize not only on the potentials of paraga to increase the crash risks of users on the road, but on the health hazards of paraga as well. Finally, the Federal Road Safety Commission (FRSC), which was formed to prevent and minimize accidents on the highways as well as the Nigerian Police, should be empowered to carry out routine alcohol breath checks on the road.

The National Agency for Food and Drug Administration and Control (NAFDAC), which the Federal Government of Nigeria has saddled with the responsibility for the control and regulation of the manufacture, sale and packaging of food and drug (including herbal remedies and alcoholic drinks) should rise up to the challenges of regulating the production, distribution and sales of paraga in the country.

## Competing interests

The authors declare that they have no competing interests.

## Authors' contributions

OSK designed the study, executed the data management, and drafted the manuscript. AEA analyzed the samples and revised the manuscript. Both authors read and approved the final manuscript.
